# Endoscopic Ultrasound for Early Diagnosis of Pancreatic Cancer

**DOI:** 10.3390/diagnostics9030081

**Published:** 2019-07-24

**Authors:** Takeichi Yoshida, Yasunobu Yamashita, Masayuki Kitano

**Affiliations:** Department of Gastroenterology, Wakayama Medical University, 811-1 Kimiidera, Wakayama-city, Wakayama 641-0012, Japan

**Keywords:** endoscopic ultrasound, contrast-enhanced endoscopic ultrasound, pancreatic cancer

## Abstract

Detection of small pancreatic cancers, which have a better prognosis than large cancers, is needed to reduce high mortality rates. Endoscopic ultrasound (EUS) is the most sensitive imaging modality for detecting pancreatic lesions. The high resolution of EUS makes it particularly useful for detecting small pancreatic lesions that may be missed by other imaging modalities. Therefore, EUS should be performed in patients with obstructive jaundice in whom computed tomography (CT) or magnetic resonance imaging (MRI) does not identify a definite pancreatic lesion. Interest in the use of EUS for screening individuals at high risk of pancreatic cancer, including those with intraductal papillary mucinous neoplasms (IPMNs) and familial pancreatic cancer is growing. Contrast-enhanced EUS can facilitate differential diagnosis of small solid pancreatic lesions as well as malignant cystic lesions. In addition, EUS-guided fine needle aspiration can provide samples of small pancreatic lesions. Thus, EUS and EUS-related techniques are essential for early diagnosis of pancreatic cancer.

## 1. Introduction

Improving the prognosis of patients with pancreatic cancer is a challenge. Overall, pancreatic cancer has one of the worst prognoses among all cancers [1,2]; however, the prognosis is better if cancer is detected at an early stage. For example, patients with pancreatic cancers ≤1 cm in size at the time of diagnosis have a 5-year survival rate of 80.4% [3]. Because such small cancers now account for 0.8% of all pancreatic cancers [3], detection of more small cancers would contribute to improving mortality rates.

**Figure 1:**
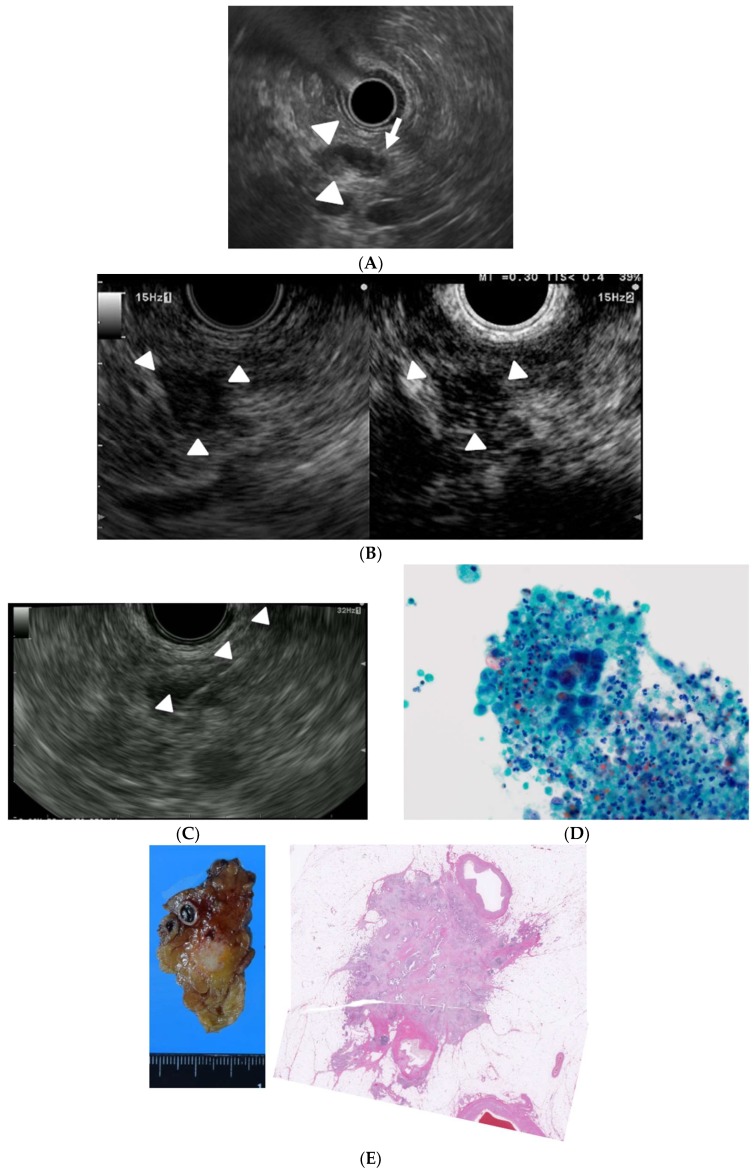
A case of small pancreatic cancer. (**A**) Endoscopic ultrasound (EUS) detection of a hypoechoic lesion (9 mm, pancreatic body, arrowhead) with distal dilation of the pancreatic duct (arrow); (**B**) Conventional EUS showing a hypoechoic lesion (arrowhead) at the pancreas body (left) and contrast-enhanced EUS (CE-EUS) showing the lesion has a lower intensity than that of the surrounding tissue (right); (**C**) EUS-guided fine-needle aspiration (EUS-FNA). The needle targeted the lesion through the stomach (arrowhead); (**D**) Cytological examination of the aspirated material suggested the presence of an adenocarcinoma (Papanicolaou ×1000); (**E**) The surgically removed lesion (left) and histological findings revealed a final diagnosis of pancreatic cancer (right).

Endoscopic ultrasound (EUS), in which the tip of the endoscope contains a high-frequency transducer, provides high resolution images of the pancreas. Indeed, its high resolution in experienced hands enables detection of focal lesions as small as 2–5 mm [4]. In addition, EUS-related techniques such as contrast-enhanced EUS (CE-EUS) and EUS-guided fine needle aspiration (EUS-FNA) are used for differential diagnosis of pancreatic lesions. Hence, EUS and EUS-related techniques are essential modalities for diagnosis of pancreatic cancers (Figure 1A–E).

Here, we review the current literature with respect to the role of EUS for the diagnosis of pancreatic cancers, particularly small pancreatic cancers.

## 2. EUS for Detection of Small Pancreatic Lesions

Data from a large number of studies reveal that EUS is the most sensitive imaging modality for detection of pancreatic lesions. Overall, the sensitivity of EUS for detecting pancreatic tumors is 94% (*n* = 1170), which is consistently higher than that of computed tomography (CT) and transabdominal ultrasound (US) [5].

Although there are limited studies regarding the detection of small pancreatic lesions using EUS, this technique seems to be more useful than other imaging modalities [6,7,8,9,10,11,12]. The sensitivity of EUS, CT, and magnetic resonance imaging (MRI) for detecting lesions <30 mm in diameter is 93%, 53%, and 67%, respectively (*n* = 49) [6]. The sensitivity of EUS for detecting pancreatic tumors ≤20 mm in diameter is higher than that of contrast-enhanced CT (94.4% vs. 50.0%, respectively; *n* = 36) [7], whereas the sensitivity of EUS for detecting pancreatic cancers ≤10 mm in diameter is higher than that of US, CT, and positron emission tomography (PET) (>80% vs. 17–70%, 33–75%, and 50%, respectively) [13]. Of note, EUS can detect pancreatic tumors not identified by other modalities [9,10,11,12]. A meta-analysis summarizing four studies (*n* = 206) reported that the sensitivity and specificity of EUS for detecting pancreatic tumors that were indeterminate on multidetector CT (MDCT) were 85% and 58%, respectively [14].

In clinical practice, a normal pancreas depicted by EUS essentially rules out pancreatic cancer. However, follow-up with EUS or another imaging modality is needed for patients that present with chronic pancreatitis without a definite mass, for patients with a diffusely infiltrating carcinoma, or for patients with a recent episode (<4 weeks) of acute pancreatitis [15,16,17].

## 3. EUS for Surveillance of Asymptomatic High Risk Subjects

Because EUS provides high resolution images, it would seem reasonable to use it to screen asymptomatic high risk cohorts for pancreatic cancer, including those with premalignant pancreatic lesions (i.e., intraductal papillary mucinous neoplasms (IPMNs)) and those with familial pancreatic cancer [18].

One study has evaluated the utility of EUS for detecting early pancreatic cancers during surveillance of patients with IPMN [19]. The authors screened 102 patients with branch-duct IPMN with EUS over a median period of 3.5 years; seven IPMN-concomitant pancreatic cancers were identified. EUS identified 100% of the seven IPMN-concomitant cancers, whereas MDCT, MRI, and US identified only 56%, 60%, and 39% of those lesions, respectively. The mean size of the pancreatic tumors was 16 mm, and 85.7% (6/7) of patients underwent surgery. Thus, frequent follow-up with EUS would enable early detection of pancreatic cancer in patients with IPMNs.

Patients with hereditary and familial pancreatic cancer are another high risk group. Canto et al. screened such individuals and made a blinded comparison of images obtained from CT, MRI, and EUS; they found that EUS was more sensitive (42%) for detecting pancreatic abnormalities than CT (11%) and MRI (33%) [20].

## 4. EUS for Differential Diagnosis of Small Pancreatic Lesions

CE-EUS is very useful for differential diagnosis of pancreatic lesions. The technique is based on the fact that microbubbles in the contrast agents are disrupted by ultrasound waves, resulting in signals that are detected by the ultrasound imager. A meta-analysis (*n* = 887) revealed that the pooled sensitivity and specificity of CE-EUS were 93% and 80%, respectively [21]. In contrast, the sensitivity and specificity of EUS alone were 95% and 53% (*n* = 115) [22].

Accurate diagnosis of small pancreatic lesions is very important because a relatively high proportion of these lesions are not pancreatic cancers [23]. Very few studies have compared the use of different imaging modalities for differential diagnosis of small pancreatic lesions. However, one study has reported that CE-EUS is particularly useful in this case [8]. The authors provide a detailed description of the diagnostic performance of CE-EUS. With respect to small pancreatic lesions (<2 cm in diameter, *n* = 67), the sensitivity and specificity of CE-EUS were 91.2% and 94.4%, respectively. The corresponding values of MDCT were 70.6% and 91.9%, respectively (*p* < 0.05).

CE-EUS is also useful for differential diagnosis of malignant pancreatic cystic lesions. For example, nodules in cysts associated with IPMN imply pancreatic cancer, although distinguishing mural nodules from mucous clots can be difficult. In this respect, the sensitivity and specificity of CE-EUS are 100% and 80–97%, respectively, while contrast-enhanced MDCT achieves values of 58–71% and 100%, respectively [24,25]. Additionally, evaluating mural nodules with CE-EUS is useful for estimating the malignant potential of IPMNs [26,27].

Needle-based confocal laser endomicroscopy (nCLE), which provides real-time in vivo imaging of structures, enables the observation of the inner wall of pancreatic cystic lesions during an EUS-FNA procedure. The sensitivity and specificity of nCLE for the diagnosis of pancreatic cystic lesions are 95–98% and 94–100%, respectively [28,29]. It has the potential to play a complementary role in the differential diagnosis of cystic pancreatic lesions. Additionally, more new technologies for contrast enhanced agents have been developed. In mice models, contrast agents specific to tumor vascular marker protein can acquire ultrasound molecular imaging of cancer lesions [30,31]. These promising technologies would help EUS to detect early pancreatic cancers among pancreatic cystic lesions.

## 5. EUS for Acquisition of Tissue from Small Pancreatic Tumors

EUS-FNA is the standard method for obtaining tissue samples from pancreatic lesions because this method has few complications. Data from four meta-analyses suggest that the sensitivity and specificity of EUS-FNA for diagnosis of pancreatic cancer are 85–92% and 96–98%, respectively [32,33,34,35]. The sensitivity of EUS-FNA for pancreatic cancer in patients with a negative or non-diagnostic sample from a previous endoscopic retrograde cholangiopancreatography (ERCP) exceeds 90% [36].

Few reports have examined the diagnostic performance of EUS-FNA with respect to small pancreatic lesions. Its sensitivity and specificity for pancreatic lesions >10 and ≤20 mm in size are 75.9–92.0% and 93.8–100%, respectively, whereas those for pancreatic lesions ≤ 10 mm in size are 40.0–100% and 80–100%, respectively [37,38,39]. A few studies have tried to examine the correlation between the performance of EUS-FNA and lesion size, but the data are contradictory [37,38,39,40,41].

By contrast, clinical practice guidelines from the National Comprehensive Cancer Network (NCCN) and the European Society for Medical Oncology (ESMO), along with a consensus statement by the International Study Group of Pancreatic Surgery, propose that proof of malignancy from a biopsy is not required for removal of early resectable pancreatic cancers [42,43,44]. Surgical resection should not be delayed when there is high clinical suspicion of pancreatic cancer. However, the definition of “high clinical suspicion of pancreatic cancer” seems obscure. Nearly 10% of resected specimens considered preoperatively as pancreatic cancer turn out to be other types of lesions, such as focal chronic pancreatitis, autoimmune pancreatitis, pancreatic tuberculosis, and pancreatic lymphoma [45,46]. New fine needle biopsy devices have been developed to improve tissue acquisition, and three types of fine needle biopsy (FNB) needles are currently available [47,48,49,50,51,52]. Needles with reverse bevel design (ProCore, Cook Medical, Bloomington, Indiana) have resulted in better histologic evaluation (81.1% vs. 69.4%, *p* = 0.048) [49] or a smaller number of passes required for diagnosis [50] than the standard FNA needles. Needles with a Franseen tip design (Acquire, Boston Scientific Corp, Natick, MA, USA) and those with a fork-tip design (SharkCore, Medtronic, Minneapolis, MN, USA) can provide larger samples than that of standard needles [51,52], and the performance of those two were comparable in yielding histologic tissue [48]. Additionally, micro-forceps through an EUS 19-guage needle might be promising for tissue acquisition in pancreatic cystic lesions [53]. Taking into consideration overall mortality and morbidity after pancreatic surgery [54], differential diagnosis using CE-EUS is very important to ensure that patients do not undergo unnecessary surgery. Additionally, because small pancreatic lesions are often difficult to identify and target, CE-EUS might help to identify the EUS-FNA target in small pancreatic lesions as well as large ones [8,55].

## 6. Conclusions

EUS is an essential modality for identifying early pancreatic cancer. CE-EUS improves characterization of pancreatic lesions detected on EUS, and EUS-FNA can confirm a pancreatic tumor with high sensitivity and specificity.
